# Long-Term Outcomes After Supravalvar Aortic Stenosis Repair: Impact of Residual Haemodynamics on Reintervention

**DOI:** 10.1093/icvts/ivag230

**Published:** 2026-08-17

**Authors:** Nabil Hussein, Branko Mimic, Mats Synnergren, Nagarajan Muthialu, Victor Tsang, Martin Kostolny

**Affiliations:** Paediatric Cardiothoracic Surgery, Great Ormond Street Hospital, London, WC1N 3JH, United Kingdom; Paediatric Cardiothoracic Surgery, Great Ormond Street Hospital, London, WC1N 3JH, United Kingdom; Paediatric Cardiothoracic Surgery, Great Ormond Street Hospital, London, WC1N 3JH, United Kingdom; Paediatric Cardiothoracic Surgery, Great Ormond Street Hospital, London, WC1N 3JH, United Kingdom; Paediatric Cardiothoracic Surgery, Great Ormond Street Hospital, London, WC1N 3JH, United Kingdom; Paediatric Cardiothoracic Surgery, Great Ormond Street Hospital, London, WC1N 3JH, United Kingdom; Institute of Cardiovascular Science, University College London (UCL), London, WC1E 6DD, United Kingdom

**Keywords:** supravalvar aortic stenosis, reintervention, Williams syndrome, left ventricular outflow tract, coronary intervention, aortic valve

## Abstract

**Objectives:**

Surgical repair of supravalvar aortic stenosis (SVAS) provides excellent early outcomes; however, late reintervention remains common. The determinants of left ventricular outflow tract (LVOT) failure, particularly the role of residual haemodynamics, remain incompletely defined. We sought to identify predictors of LVOT reintervention in a contemporary cohort.

**Methods:**

A retrospective study of consecutive patients aged <18 years undergoing SVAS repair between 2005 and 2024 was performed. Primary end-points were overall survival and freedom from LVOT reintervention. Survival and freedom from reintervention were assessed using Kaplan-Meier analysis. Multivariable Cox regression was used to identify independent predictors of LVOT reintervention.

**Results:**

Seventy-seven patients underwent repair at a median age of 3 (1-8) years, with median follow-up of 9.3 (6-14) years. There was no early or late mortality; 19 patients (25%) required reintervention, including 16 (21%) LVOT-related procedures. Freedom from LVOT reintervention was 74% at 10 years and 71% at 15-20 years. Discharge LVOT velocity >3 metres per second (m/s) was independently associated with LVOT reintervention (hazard ratio [HR] 11.66, 95% confidence interval [CI] 3.56-38.18; *P* < .001). Younger age was also independently associated with increased risk (HR per year 0.74, 95% CI, 0.59-0.94; *P* = .013) for LVOT reintervention. Coronary abnormalities were documented in 17% of patients, with one early coronary reintervention.

**Conclusions:**

Surgical repair of supravalvar aortic stenosis is associated with excellent long-term survival. Residual discharge velocity emerged as an independent predictor of late LVOT reintervention, supporting the importance of achieving optimal haemodynamic relief at the index operation and structured lifelong surveillance.

## Graphical abstract

**Figure:**
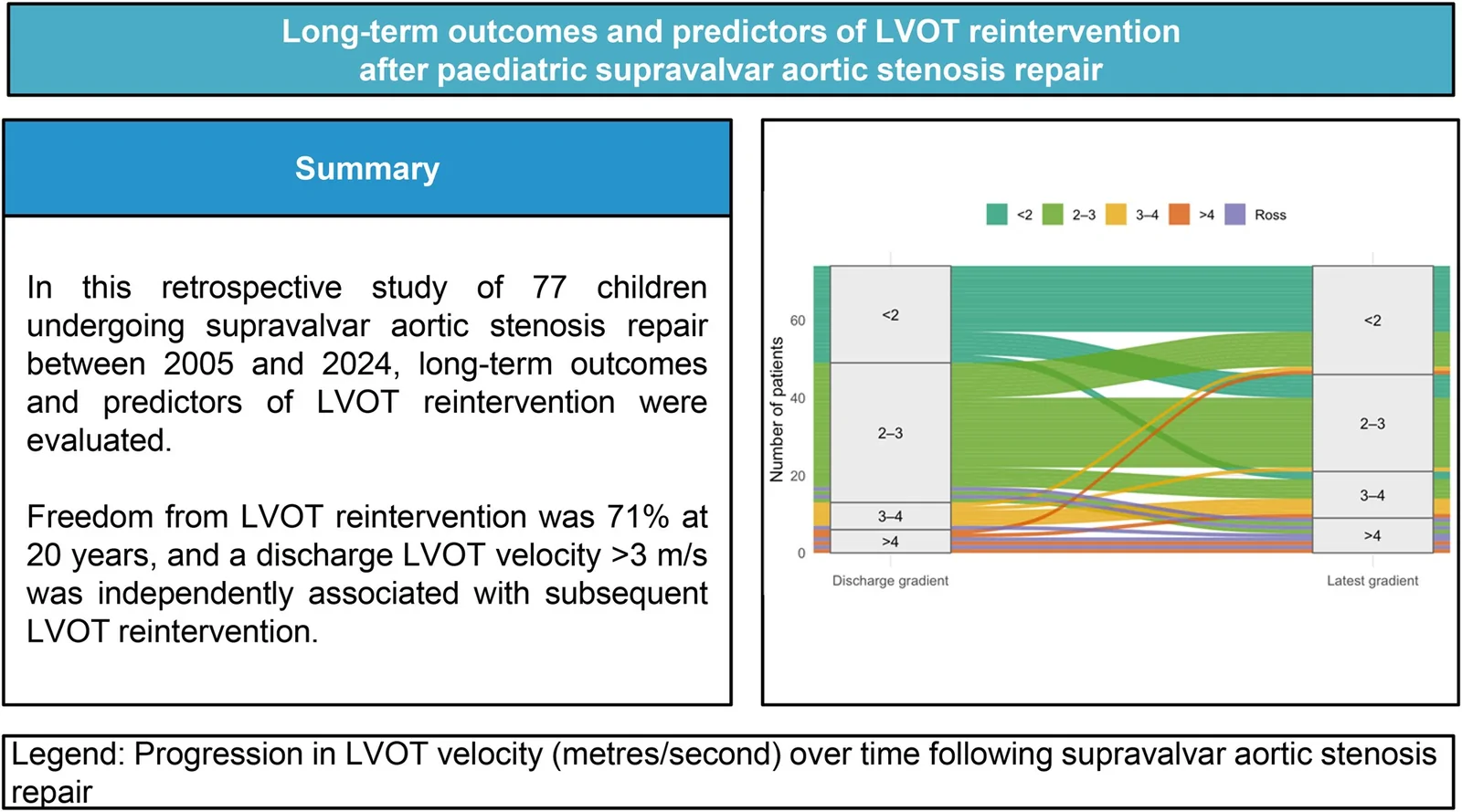

## INTRODUCTION

Supravalvar aortic stenosis (SVAS) is an uncommon congenital arteriopathy characterized by narrowing at the sinotubular junction and frequently associated with Williams syndrome or elastin-related arteriopathy. Surgical repair is the treatment of choice for significant obstruction and is typically performed in childhood. Advances in surgical technique and perioperative care have resulted in excellent contemporary survival, with long-term survival exceeding 90% in modern series.^1–3^

Despite favourable survival, reintervention remains a substantial long-term burden. Late procedures, most commonly involving the left ventricular outflow tract (LVOT), occur in approximately 20%-30% of patients during extended follow-up.^2^^,^^3^ Younger age at repair and diffuse arteriopathy have been identified as potential risk factors for reoperation^1^^,^^4^; however, the relative contribution of operative strategy versus residual haemodynamic obstruction remains incompletely defined.

Coronary artery involvement is increasingly recognized in SVAS and may influence both perioperative risk and long-term outcomes.^5^ In addition, the impact of associated right-sided lesions and the necessity for concomitant intervention remain areas of ongoing debate.^2^ Contemporary data clarifying the drivers of late LVOT failure and the clinical significance of coronary pathology are therefore required.

The present study aimed to evaluate long-term outcomes following SVAS repair in a contemporary single-centre cohort, with particular focus on (1) survival and reintervention burden and (2) identifying clinical factors associated with late LVOT reintervention.

## METHODS

### Patients and methods

This was a retrospective cohort study of consecutive patients aged <18 years undergoing surgical repair of SVAS between January 2005 and December 2024 at a single tertiary referral centre.

All patients undergoing primary SVAS repair during the study period were eligible for inclusion.

Patients with prior surgical intervention involving the LVOT, including coarctation repair, subaortic resection, balloon aortic valvuloplasty, surgical aortic valvotomy, arch repair, or pulmonary artery reconstruction, were included. Patients with previous cardiac operations not involving the LVOT (including total anomalous pulmonary venous connection repair, interrupted aortic arch repair, or arterial switch operation) were excluded. For this study, LVOT will refer to the area, including the aortic subvalvar, valve, and/or supravalvar region including aortic arch.

Patients were identified through institutional surgical databases. Baseline demographic characteristics, genetic diagnosis, presence of right-sided lesions, and prior surgical history were collected retrospectively from electronic hospital records (**Table 1**). All patients underwent preoperative echocardiography and computed tomography angiography to define the extent of stenosis and assess coronary anatomy, which is particularly important in patients with SVAS given the recognized perioperative anaesthetic risk associated with coronary involvement and myocardial ischaemia.^6^ Operative details and postoperative course were obtained from operative reports and inpatient records (**Table 2**).

**Table 1.** Baseline Characteristics of Patients Undergoing Supravalvar Aortic Stenosis Repair (*n* = 77)

Right-sided lesion at time of surgery	28 (36)
Age at surgery, years	3 (1-7)
Weight, kg	13.2 (8-22)
Male	50 (65%)
Genetics diagnosis	
Williams syndrome	35 (45)
Familial elastin arteriopathy	5 (6)
None	37 (48)
Right-sided lesion address at index operation (*n* = 28)	7 (25%)
Previous surgery	13 (17)

Data represented as median (interquartile range) or *n* (%).

**Table 2.** Operative Characteristics

Repair technique	
1 sinus	29 (38)
2 sinus	28 (36)
Doty repair	15 (19)
3 sinus	20 (26)
Patch material	
Bovine pericardium	41 (53)
Homograft	26 (34)
Autologous pericardium	5 (6)
Cardiocel	4 (5)
Dacron	1 (1)
Concomitant procedure	19 (25)
Aortic valvotomy/valvuloplasty	7 (9)
Aortic arch repair	3 (4)
Subaortic resection	4 (5)
Pulmonary valvotomy	2 (3)
Pulmonary arterioplasty	4 (5)
Cor triatriatum repair	1 (1)
VSD closure	1 (1)
Coronaries	
Normal	64 (83)
Abnormal coronaries	13 (17)
Abnormal coronary classification (*n* = 13)	
Type 1—Ostial ridge	8 (62)
Type 2—Leaflet fusion	0
Type 3a—Ostial hypoplasia	3 (23)
Type 3b—Ostial and artery hypoplasia	0
Other	
Anomalous coronary origin	1 (8)
High coronary origin	1 (8)
Osteoplasty at time of index operation	0
Osteoplasty postoperative	1 (1)
CPB time (median/IQR)	91 (60-113)
Cross-clamp time (median/IQR)	59 (36-75)
Early postoperative outcomes	
Length of hospital stay, days	5(3-9)
Major postoperative complication	8 (10)
ECMO	5 (6)
ECMO duration, days	5(5-7)
Dialysis	1 (1)
Unplanned reoperation	2 (3)

Data represented as median (interquartile range) or *n* (%).

### Operative technique and coronary assessment

Surgical repair of supravalvar aortic stenosis was performed using either a 1-, 2-, or 3-sinus reconstruction technique, according to anatomical considerations and surgeon preference.^7–9^ Therefore, analyses involving operative technique were exploratory and intended to identify associations with late outcomes rather than directly compare strategies. Regardless of the technique employed, reconstruction involved patch augmentation of the supravalvar region, including the sinotubular junction and ascending aorta, with enlargement of at least one sinus of Valsalva. The distinction between techniques related primarily to the number of sinuses reconstructed rather than the overall extent of ascending aorta augmentation.

Concomitant procedures involving the subaortic region, aortic valve, aortic arch, pulmonary arteries, or right-sided structures were recorded. Coronary anatomy was assessed intraoperatively. Coronary lesions were classified according to previously described criteria and included: (1) ostial ridge; (2) leaflet fusion; and (3) complex lesions, subdivided into (3a) ostial hypoplasia and (3b) combined ostial and proximal coronary artery hypoplasia.^10^ Coronary interventions at the index operation or during follow-up were documented.

Primary end-points included overall survival and freedom from LVOT reintervention. Left ventricular outflow tract reintervention was defined as any subsequent surgical or catheter-based procedure involving the LVOT, including repeat SVAS repair, subaortic resection, aortic arch repair, aortic valve repair or replacement, or Ross procedure. Secondary end-points included freedom from any reintervention and the occurrence of coronary interventions. Major postoperative morbidity was defined as the occurrence of cardiac arrest, neurological injury, requirement for extracorporeal membrane oxygenation (ECMO), renal impairment requiring dialysis, or unplanned reoperation during the index admission.

Follow-up data were obtained from hospital clinical records and serial echocardiography, from hospital discharge to the latest follow-up. Clinical outcomes and interval reinterventions were recorded.

The study was registered with the institution’s clinical audit department; however, in accordance with UK NHS National Research Ethics Service guidance, neither individual informed consent nor formal research ethics committee review was required, as the study was undertaken by the direct clinical team using information previously collected during routine patient care.

### Statistical analysis

Continuous variables are presented as median (interquartile range [IQR]) owing to non-normal distributions and categorical variables as *n* (%). Overall survival and freedom from reintervention were estimated using the Kaplan-Meier method and compared using the log-rank test. Multivariable Cox proportional hazards regression was performed to identify independent predictors of LVOT reintervention. Clinically relevant variables were selected a priori, including discharge LVOT velocity (>3 metres/second [m/s] vs ≤3 m/s), age at surgery (per year increase), prior isolated LVOT surgery, and concomitant aortic valve surgery. Hazard ratios (HRs) are reported with 95% confidence intervals (CIs). Exploratory analysis of operative technique, Williams syndrome, and right-sided lesions were evaluated using Kaplan-Meier analysis and log-rank testing. Coronary interventions were analysed descriptively due to limited event numbers. Analyses were performed using complete-case data using R statistical software (R Foundation for Statistical Computing, Vienna, Austria), with *P* < .05 considered statistically significant.

## RESULTS

Over the 20-year period, 77 consecutive patients underwent surgical repair of SVAS at a median age of 3 (1-8) years and median weight of 13 (8-23) kg. Fifty (65%) patients were male. Thirty-five (45%) had Williams syndrome and 5 (6%) had a familial elastin arteriopathy. Twenty-eight (36%) patients had a right-sided lesion at the time of surgery, with 7 (25%) of these operated on during the repair. The sinus techniques used were similar across the study period, with bovine pericardium the most used patch material (*n* = 41, 53%) followed by homograft (*n* = 26, 34%). Median cardiopulmonary bypass and aortic cross-clamp times were 91 (60-113) and 59 (36-75) min, respectively. Median follow-up was 9.3 (6-14) years. Complete follow-up was available in 74 patients (96%), with 3 patients lost to follow-up. Three patients had missing discharge LVOT velocity measurements and were excluded from analyses involving discharge haemodynamics.

### Survival and morbidity

Overall survival following SVAS repair was 100% at 20 years. Major postoperative morbidity occurred in 8 patients. Five patients required ECMO, with a median duration of 5 (5-7) days; all patients were successfully weaned. Two patients underwent coronary angiography while on ECMO with severe proximal left coronary artery (LCA) stenosis identified in one patient. One patient required dialysis for acute renal impairment, and 2 patients underwent unplanned reoperation during the index admission.

### Reinterventions

During follow-up, 19 of 77 patients (25%) underwent at least one reintervention. The patient with LCA narrowing underwent coronary osteoplasty during their hospital stay. Other reinterventions included arch repair (*n* = 6), repeat SVAS repair (*n* = 5), Ross procedure (*n* = 3), aortic valve repair (*n* = 2), subaortic resection (*n* = 1), balloon pulmonary angioplasty (*n* = 1), and mitral valve repair (*n* = 1). Among the 19 patients requiring reintervention, 6 (32%) had undergone previous cardiac surgery and 10 (53%) underwent a concomitant LVOT procedure at the index operation, including 4 (21%) who underwent concomitant arch repair. Sixteen patients (21%) underwent LVOT-related reintervention. Freedom from LVOT reintervention was 88.6% at 1 year, 83.8% at 5 years, and 74.2% at 10 years. At 15 years, freedom from LVOT reintervention was 71.0%, remaining stable at 20 years. Left ventricular outflow tract reinterventions occurred predominantly within the first decade following surgery (**Figure 1A**).

**Figure 1.**
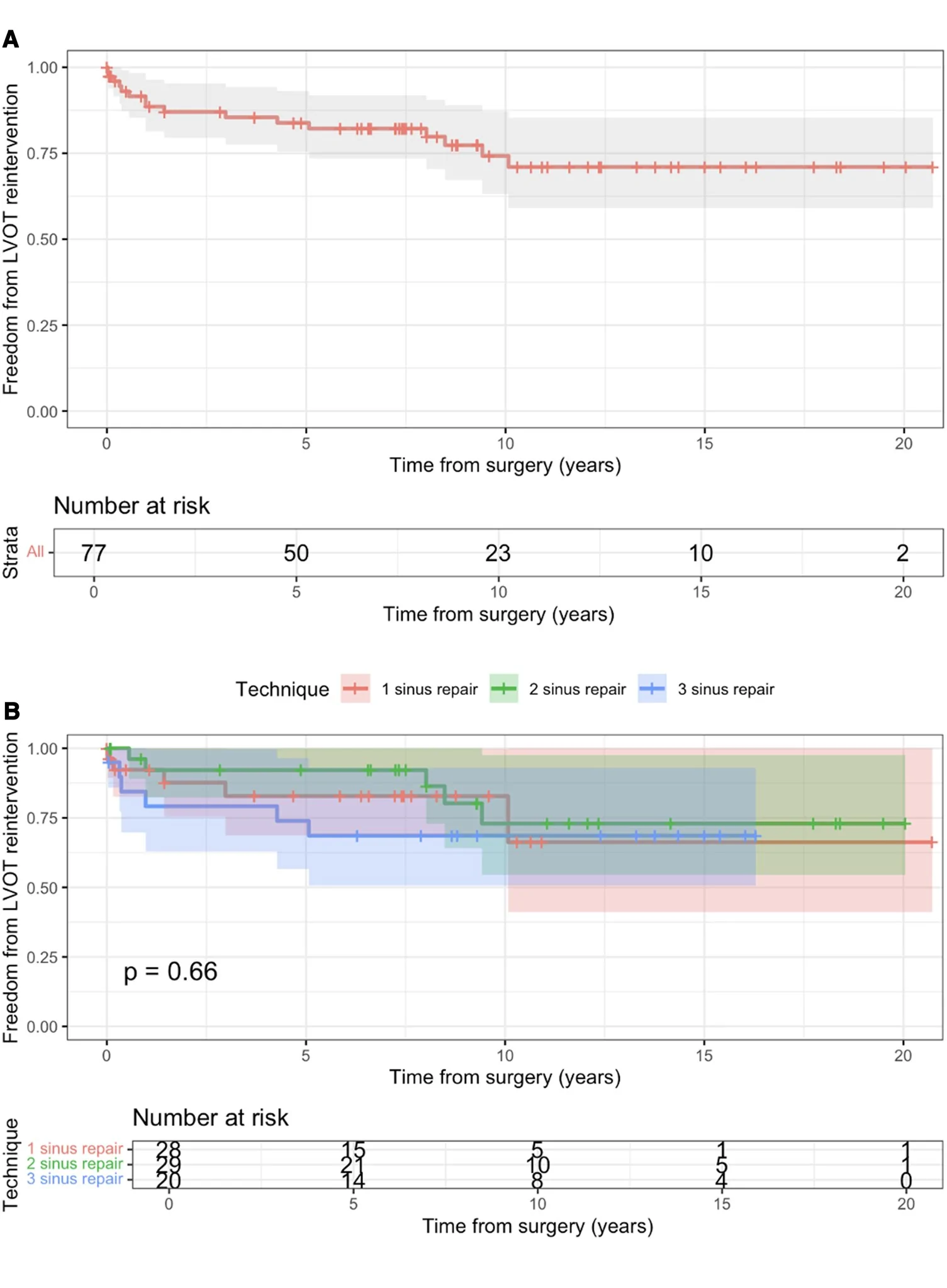
(A) Freedom from left ventricular outflow tract (LVOT) reintervention following supravalvar aortic stenosis repair was 88.6% at 1 year, 83.8% at 5 years, and 74.2% at 10 years. At 15 years, freedom from LVOT reintervention was 71.0%, remaining unchanged at 20 years. Left ventricular outflow tract reinterventions occurred predominantly within the first decade following surgery, with a gradual decline in freedom from LVOT reintervention over time. (B) Kaplan-Meier analysis demonstrated no significant difference in freedom from LVOT reintervention according to surgical technique over the entire follow-up period (*P* = .66)

To further characterize the mechanisms underlying LVOT reintervention, the anatomical level and haemodynamic profile of patients requiring reintervention were examined. Further analysis of the 16 patients requiring LVOT-related reintervention demonstrated distinct patterns according to the anatomical level of obstruction. Reinterventions involving the aortic arch (*n* = 6) or repeat supravalvar repair (*n* = 5) were typically performed for obstruction distal to the original repair site. Most patients undergoing arch reintervention had Williams syndrome (4/6), whereas only one patient undergoing repeat SVAS repair had Williams syndrome.

In contrast, patients requiring aortic valve intervention demonstrated gradients localized to the valvar level. Two patients underwent aortic valve repair and 3 underwent Ross procedure, none of whom had Williams syndrome. Nearly all patients (4/5) had a commissurotomy at the time of the index operation. In all patients undergoing LVOT reintervention, the discharge velocity exceeded 3 m/s at the anatomical level corresponding to the subsequent site of reintervention.

### Subgroup analyses

#### Surgical technique and coronary findings

Freedom from LVOT reintervention did not differ significantly according to surgical technique. Kaplan-Meier analysis demonstrated similar freedom from LVOT reintervention across techniques throughout follow-up, with no significant difference between groups over the entire follow-up period (log-rank *P* = .66) (**Figure 1B**). Pairwise comparisons between techniques were also non-significant. Coronary abnormalities were documented in 13 patients (17%). These included ostial ridge (*n* = 8), ostial hypoplasia (*n* = 3), anomalous coronary origin (*n* = 1), and high coronary origin (*n* = 1). No cases of leaflet fusion or combined ostial and proximal coronary artery hypoplasia were identified during index surgery. One patient required early reoperation for a coronary-related issue. Coronary ostial ridge was routinely assessed at the index operation and addressed if required.

#### Williams syndrome and right-sided lesions

In patients without Williams syndrome, freedom from any reintervention was 92.3% at 1 year, 81.6% at 5 years, and 67.4% at 10 years (**Figure 2A**). Patients with Williams syndrome demonstrated lower early freedom from reintervention (80.6% at 1 year and 77.0% at 5 years), which remained stable thereafter. However, these differences were not statistically significant (*P* = .84). Likewise, freedom from any reintervention did not differ significantly according to the presence of a right-sided lesion (*P* = .7) (**Figure 2B**).

**Figure 2.**
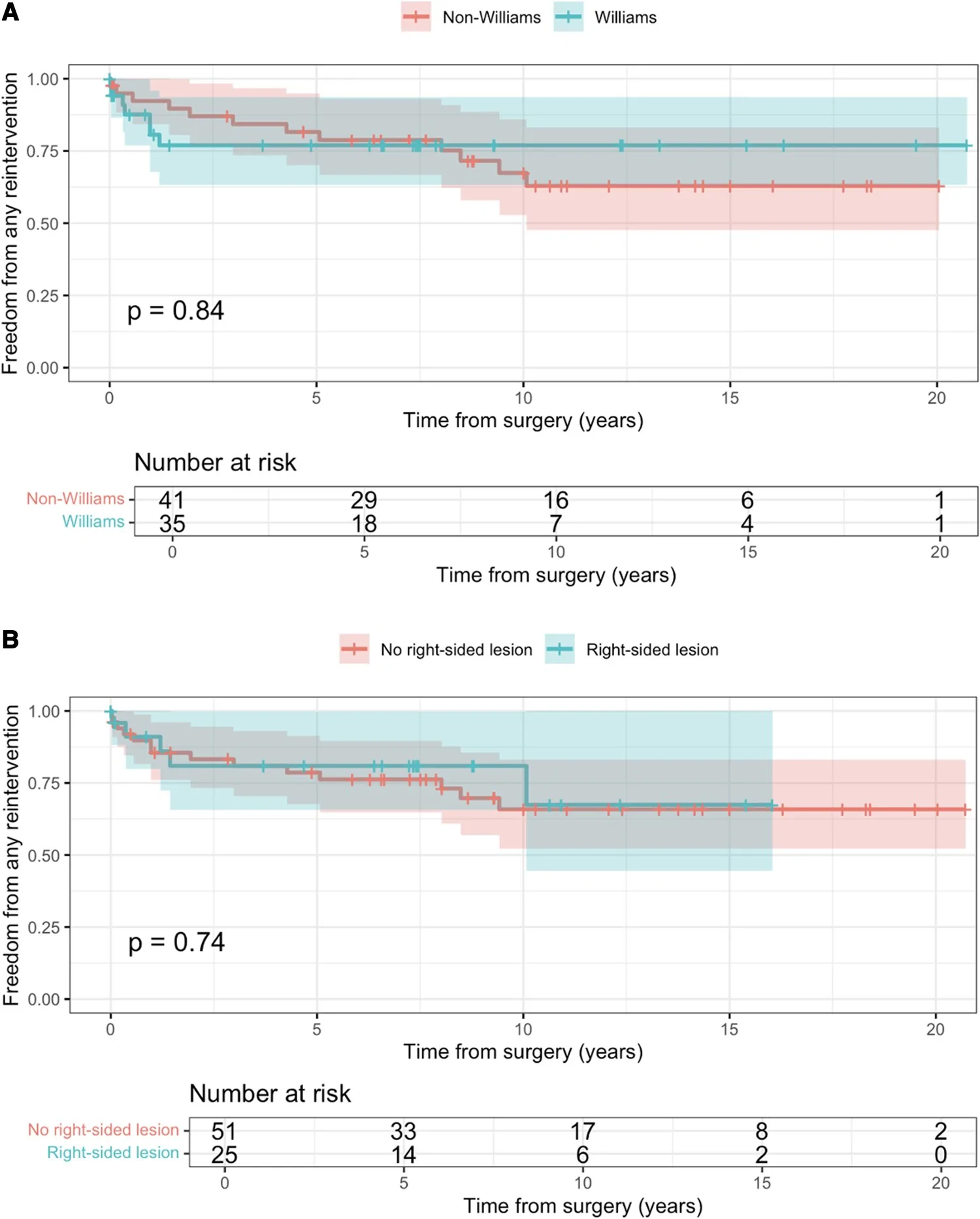
Comparison of freedom from intervention between Williams vs non-Williams (A) and the presence or absence of a right-sided lesion at the time of supravalvular aortic stenosis repair (B). There were no statistically significant differences, *P* > .5

#### Concomitant aortic valve surgery and discharge LVOT gradient

Freedom from LVOT reintervention was associated with concomitant aortic valve surgery at the index operation (*P* = .04), with lower freedom from LVOT reintervention observed in patients undergoing aortic valve surgery (**Figure 3A**). However, this finding should be interpreted cautiously given the small number of patients (*n* = 8) and limited numbers at risk during late follow-up.

**Figure 3.**
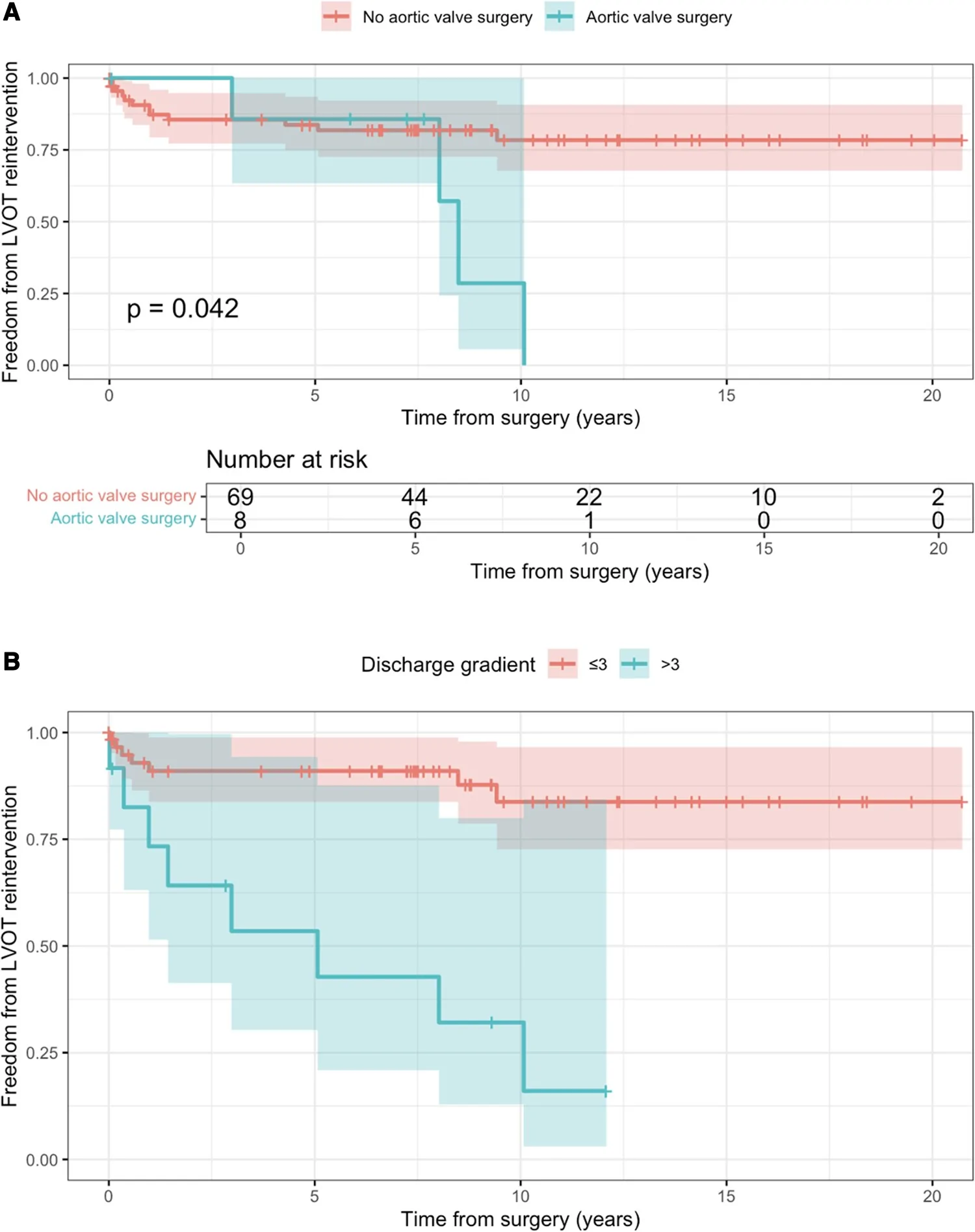
(A) Patients undergoing aortic valve surgery demonstrated lower freedom from LVOT reintervention over time compared with those not requiring aortic valve intervention (*P *= .04). (B) Patients with a discharge LVOT velocity >3 m/s demonstrated significantly lower freedom from LVOT reintervention compared with those with lower velocities. Abbreviation: LVOT, left ventricular outflow tract

Median discharge LVOT velocity was 2.1 (1.7-2.7) m/s, which remained the same at latest follow-up. Patients with a discharge velocity >3 m/s demonstrated significantly lower freedom from LVOT reintervention compared with those with lower velocities (*P* < .001). Receiver operating characteristic analysis demonstrated good discrimination for subsequent LVOT reintervention (AUC 0.77, 95% CI, 0.61-0.94). A threshold of >3 m/s yielded a sensitivity of 60% and specificity of 93%. Kaplan-Meier curves demonstrated early separation with progressive divergence over time (**Figure 3B**). In patients with discharge velocity ≤3 m/s, freedom from LVOT reintervention was 91% at 1 and 5 years and 83.8% at 10 years. In contrast, patients with discharge velocity >3 m/s had freedom from LVOT reintervention of 73.3% at 1 year, 53.5% at 5 years, and 32.1% at 10 years. **Figure 4** demonstrates the progression in LVOT velocity (m/s) over time following repair.

**Figure 4.**
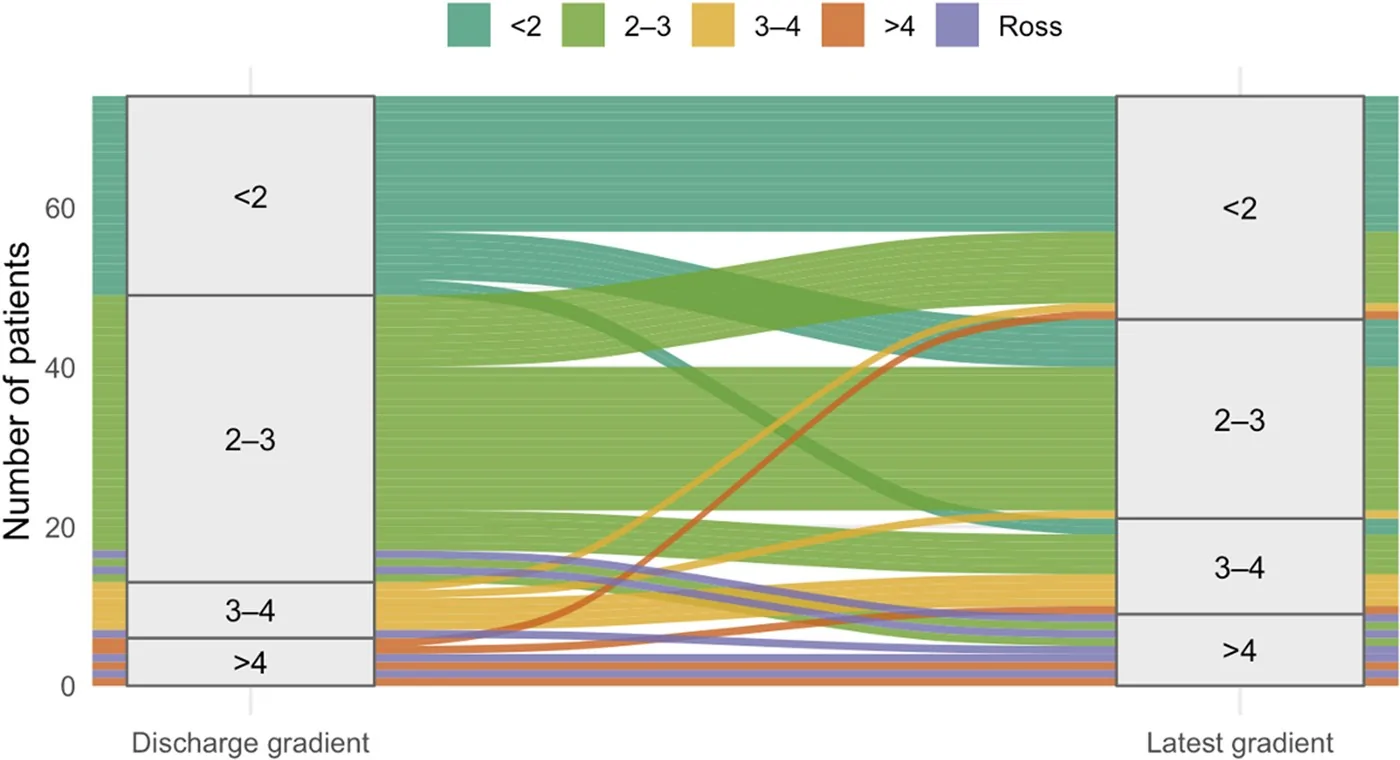
Progression in Left Ventricular Outflow Tract Velocity (Metres/Second) Over Time Following Repair, Including Patients Who Eventually Required a Ross Operation (*n* = 5)

On multivariable Cox proportional hazards analysis (**Table 3**), a discharge LVOT velocity >3 m/s was independently associated with a markedly increased risk of LVOT reintervention (HR 11.66, 95% CI, 3.56-38.18; *P* < .001). Younger age at surgery was also independently associated with increased risk of LVOT reintervention (HR per year increase 0.74, 95% CI, 0.59-0.94; *P* = .013). After adjustment, concomitant aortic valve surgery and prior isolated LVOT surgery were not independently associated with LVOT reintervention.

**Table 3.** Univariable and Multivariable Cox Proportional Hazards Analysis for Predictors of LVOT Reintervention

Variable	Univariable HR (95% CI)	*P*-value	Multivariable HR (95% CI)	*P*-value
Discharge LVOT velocity >3 m/s	7.66 (2.75-21.29)	<.001	11.66 (3.56-38.17)	<.001
Age at surgery	0.86 (0.73-1.00)	.051	0.74 (0.59-0.94)	.014
Previous isolated LVOT surgery	2.36 (0.67-8.31)	.181	2.88 (0.48-17.37)	.249
Concomitant aortic valve surgery	3.08 (0.98-9.64)	.054	0.95 (0.23-3.94)	.939

Abbreviations: CI, confidence interval; HR, hazard ratio; LVOT, left ventricular outflow tract.

## DISCUSSION

In this 20-year single-centre experience, surgical repair of SVAS was associated with excellent long-term survival. Contemporary series similarly report survival exceeding 90% at 15-20 years,^1^^,^^2^ with pooled analyses confirming favourable long-term outcomes in the modern era.^3^ These findings contrast with earlier historical cohorts in which late mortality remained significant.^3^^,^^11^^,^^12^

Despite excellent survival, reintervention remains the principal determinant of late outcome. In the present cohort, 25% of patients required reintervention, predominantly involving the LVOT. Freedom from LVOT reintervention was 74% at 10 years and 71% at 15-20 years, closely aligning with contemporary reports.^1^^,^^2^ Multicentre analyses demonstrate cumulative reintervention rates approaching 20%-30% during long-term follow-up.^2^^,^^3^ These findings reinforce the need for structured lifelong surveillance.

A central finding of this study is the impact of residual haemodynamics. Discharge LVOT velocity >3 m/s was independently associated with an 11-fold increased risk of LVOT reintervention. Previous studies have identified younger age and anatomical severity as predictors of reoperation,^1^^,^^4^ but the specific role of immediate postoperative gradient has been less consistently evaluated. Our data suggest that the adequacy of haemodynamic relief at the index operation may be more important than the specific reconstructive strategy employed.

Residual postoperative velocity is likely multifactorial and should not necessarily be interpreted as evidence of a suboptimal repair. Patients with Williams syndrome commonly developed obstruction beyond the original repair site, consistent with diffuse elastin arteriopathy, whereas patients requiring aortic valve intervention demonstrated elevated valvar velocities reflecting intrinsic valve pathology. Residual velocity may therefore represent both disease severity and haemodynamic burden rather than technical limitations alone. Accordingly, residual velocity should not be interpreted as an isolated indication for reintervention but should prompt careful reassessment of the underlying anatomy. A discharge velocity >3 m/s identifies patients at increased risk of subsequent LVOT reintervention who warrant closer surveillance rather than immediate intervention based on a rigid haemodynamic threshold.

Given the non-random allocation of operative technique according to anatomical considerations and surgeon preference, meaningful comparisons between repair techniques cannot be made within this observational cohort. Although operative technique was explored as a potential factor associated with late outcomes, any observed differences are inherently susceptible to selection bias and residual confounding. Within these limitations, no association between surgical technique and late reintervention was demonstrated, consistent with previous reports.^2^^,^^11^ However, given the limited number of reintervention events, the study may be underpowered to detect modest technique-specific differences. Furthermore, the 3 repair techniques were used throughout the study period without a clear era effect, suggesting that changes in practice are unlikely to explain these findings. Examination of patients undergoing LVOT reintervention also suggested that the anatomical level of residual obstruction may influence the pattern of late failure.

In this cohort, distinct anatomical patterns of late failure were observed. Reinterventions involving the aortic arch or repeat supravalvar repair were most commonly observed in patients with Williams syndrome and were associated with gradients distal to the original repair site. Notably, most arch reinterventions occurred within the first year following repair (5/6 patients). Four of the 6 patients undergoing arch reintervention had Williams syndrome, consistent with the diffuse vascular involvement characteristic of elastin-related arteriopathy. Review of these cases demonstrated that 3 patients had recognized arch pathology at the time of the index operation, including one patient who underwent concomitant arch repair and 2 patients with borderline arch involvement on preoperative imaging who did not undergo arch repair at the index operation. In addition, 4 patients were discharged with elevated arch velocities (>3 m/s), suggesting residual arch obstruction following repair. These findings suggest that residual or recurrent arch obstruction contributed to the need for reintervention in the majority of cases, whereas only one patient underwent late arch intervention more than 9 years after repair. These observations emphasize the importance of careful preoperative assessment of the aortic arch, particularly in patients with Williams syndrome and suspected diffuse arteriopathy. Cross-sectional imaging may be helpful in selected patients to better define the extent of arch involvement and guide operative planning.

In contrast, patients requiring aortic valve intervention demonstrated gradients localized to the valvar level and did not have Williams syndrome, suggesting a distinct mechanism related to intrinsic valvar pathology rather than diffuse arterial disease. Notably, in all patients requiring LVOT reintervention, the discharge velocity exceeded 3 m/s at the anatomical level corresponding to the subsequent site of reintervention. These observations further reinforce the importance of residual haemodynamic obstruction as a driver of late reintervention. Although concomitant aortic valve surgery was associated with reintervention on univariable analysis, this did not persist after adjustment, likely reflecting greater baseline disease complexity. Younger age remained independently associated with reintervention, consistent with prior observations in contemporary cohorts.^1^^,^^4^ However, this relationship should be interpreted with caution as younger patients may represent a subgroup with more severe or diffuse disease requiring earlier intervention, rather than demonstrating a detrimental effect of surgery at a young age. Consequently, these findings should not be interpreted as supporting delayed intervention when clinically indicated.

Neither Williams syndrome nor the presence of right-sided lesions independently predicted reintervention. While pulmonary artery stenosis has been associated with late morbidity in multicentre studies,^2^ and reconstruction strategies in Williams syndrome have demonstrated variable long-term impact,^13^ our data did not demonstrate a significant long-term effect of right-sided pathology. These findings suggest that selective rather than routine intervention for associated right-sided lesions may be appropriate.

Coronary artery involvement in SVAS has been associated with increased perioperative risk in recent series.^5^ Although coronary abnormalities were documented in 17% of patients in the present study, only one required early coronary reintervention. Differences may reflect variation in lesion definition, indication criteria for intervention, and outcome end-points, as this study focused on long-term reintervention.^14^ While coronary anatomy remains an important perioperative consideration, it did not emerge as a factor for significant morbidity or reintervention.

Collectively, these findings suggest that residual haemodynamic obstruction is an important determinant of late outcome following SVAS repair, whereas no association between operative technique and reintervention was demonstrated within this cohort. Optimizing gradient reduction at the index operation may therefore represent the most important modifiable factor influencing long-term LVOT durability.

### Limitations

This study is limited by its retrospective single-centre design and the absence of a comparator group, which may limit the generalizability of the findings and preclude direct comparison between operative strategies. Although follow-up was excellent, with clinical follow-up available in 74 of 77 patients (96%) and a median follow-up duration of 9.3 years, the number of reintervention events was modest, limiting multivariable model complexity, reducing statistical power for subgroup analyses, and increasing the potential for residual confounding. Consequently, comparisons between operative techniques should be interpreted cautiously, as the study may be underpowered to detect modest technique-specific differences. Coronary lesion documentation was based on operative assessment and may underestimate subtle abnormalities. Echocardiographic measurements were obtained over a prolonged study period and may be subject to interobserver variability.

## CONCLUSION

Surgical repair of SVAS is associated with excellent long-term survival; however, LVOT reintervention remains an important late outcome. Residual discharge velocity emerged as an independent predictor of subsequent LVOT reintervention in this cohort. Coronary abnormalities were uncommon and rarely required intervention, but careful perioperative assessment remains important. A discharge velocity >3 m/s may help identify patients at increased risk who warrant closer postoperative surveillance, although it should not be interpreted in isolation. Optimizing haemodynamic relief at the index operation and structured lifelong surveillance are essential to maximize durability.

## AUTHOR CONTRIBUTIONS

Nabil Hussein (Conceptualization, Data curation, Formal analysis, Investigation, Methodology, Software, Validation, Visualization, Writing—original draft, Writing—review & editing), Branko Mimic (Data curation, Formal analysis, Funding acquisition, Investigation, Methodology, Project administration, Resources, Supervision, Validation, Visualization, Writing—original draft, Writing—review & editing), Mats Synnergren (Funding acquisition, Project administration, Resources, Supervision, Validation, Writing—review & editing), Nagarajan Muthialu (Funding acquisition, Project administration, Resources, Supervision, Validation, Writing—review & editing), Victor Tsang (Funding acquisition, Project administration, Resources, Supervision, Validation, Writing—review & editing), and Martin Kostolny (Conceptualization, Data curation, Formal analysis, Funding acquisition, Investigation, Methodology, Project administration, Resources, Software, Supervision, Validation, Visualization, Writing—original draft, Writing—review & editing)

## Data Availability

The data underlying this article will be shared on reasonable request to the corresponding author.

## ABBREVIATIONS

CI: confidence interval
ECMO: extracorporeal membrane oxygenation
HR: hazard ratio
IQR: interquartile range
LCA: left coronary artery
LVOT: left ventricular outflow tract
m/s: metres per second
SVAS: supravalvar aortic stenosis
